# An empirical, hierarchical typology of tree species assemblages for assessing forest dynamics under global change scenarios

**DOI:** 10.1371/journal.pone.0184062

**Published:** 2017-09-06

**Authors:** Jennifer K. Costanza, John W. Coulston, David N. Wear

**Affiliations:** 1 Department of Forestry and Environmental Resources, North Carolina State University, Research Triangle Park, North Carolina, United States of America; 2 Southern Research Station, USDA Forest Service, Blacksburg, Virginia, United States of America; 3 Southern Research Station, USDA Forest Service, Raleigh, North Carolina, United States of America; Chinese Academy of Forestry, CHINA

## Abstract

The composition of tree species occurring in a forest is important and can be affected by global change drivers such as climate change. To inform assessment and projection of global change impacts at broad extents, we used hierarchical cluster analysis and over 120,000 recent forest inventory plots to empirically define forest tree assemblages across the U.S., and identified the indicator and dominant species associated with each. Cluster typologies in two levels of a hierarchy of forest assemblages, with 29 and 147 groups respectively, were supported by diagnostic criteria. Groups in these two levels of the hierarchy were labeled based on the top indicator species in each, and ranged widely in size. For example, in the 29-cluster typology, the sugar maple-red maple assemblage contained the largest number of plots (30,068), while the butternut-sweet birch and sourwood-scarlet oak assemblages were both smallest (6 plots each). We provide a case-study demonstration of the utility of the typology for informing forest climate change impact assessment. For five assemblages in the 29-cluster typology, we used existing projections of changes in importance value (IV) for the dominant species under one low and one high climate change scenario to assess impacts to the assemblages. Results ranged widely for each scenario by the end of the century, with each showing an average decrease in IV for dominant species in some assemblages, including the balsam fir-quaking aspen assemblage, and an average increase for others, like the green ash-American elm assemblage. Future work should assess adaptive capacity of these forest assemblages and investigate local population- and community-level dynamics in places where dominant species may be impacted. This typology will be ideal for monitoring, assessing, and projecting changes to forest communities within the emerging framework of macrosystems ecology, which emphasizes hierarchies and broad extents.

## Introduction

The mix of tree species occurring in a forest community affects the ecological attributes and ecosystem services provided by forests, including biodiversity, forest stand structure, wildlife habitat, biogeochemical cycles, and water quality [1,2]. Thus, integrated assessments of forest ecosystem vulnerability, and strategies for managing or mitigating that vulnerability, require knowledge of how tree species are assembled into forest communities. On one hand, changing environmental and climate conditions have led to widespread reassembly of forest tree communities over time [3–5]. Evidence of past tree species assemblages from charcoal and pollen records indicates that at a broad scale, distributions of tree species respond individualistically to climate and environmental conditions [6,7]. Similarly, recent changes in forest species distributions are also evident, with uphill, downhill, northward and southward range shifts, as well as range expansions and contractions all documented [8–10]. Because the ranges of forest tree species are subjected to individualistic shifts over space and time, any characterization of tree species assemblages must be flexible to accommodate those shifts.

On the other hand, knowing the individual spatial distributions of tree species’ ranges is not enough to characterize forest communities even at broad extents, nor to recognize the vulnerabilities of those communities to potential future changes. Several recent studies have shown that simply overlaying individual species ranges often does not produce accurate community-level measures such as relative abundance or biomass [11,12]. In part, this is because community-level attributes depend not just on which species occur in the community, but also on the interactions among those species. Species interactions vary with the relative occurrence of those species–that is, the dominance (or conversely, the evenness) of species [13,14]. The relative occurrence or dominance of species in an ecosystem not only influences community-level attributes and species interactions, but also can be influenced by environmental change [15,16]. For example, changing climate is likely to affect the dominance structure in a community before a change in species richness is observed [13]. Therefore, a characterization of tree species assemblages that is based on recent information about the relative occurrence of species within communities will be ideal for detecting and monitoring changes in those communities as species respond jointly and individualistically to climate and environmental changes.

In addition to detection and monitoring of changes as they occur, a characterization of forest tree communities can also be a useful basis for looking forward to potential future changes. Community-level projection models and vulnerability assessments are two techniques for investigating future changes to forest communities under global change scenarios, and both rely on baseline knowledge about which communities exist in the contemporary time period. Community-level models that aim to project potential future changes in forest communities are promising because of their ability to incorporate not only environmental suitability for individual species, but also information on species interactions and other community- and population-level processes [17–19]. In a vulnerability assessment, potential *impacts* from global changes are evaluated against a community’s ability to adapt to potential global changes [20]. Impacts are a function of exposure, or the degree to which a community is likely to experience changes in a global change driver, and sensitivity, or the degree to which a community is likely to be affected by those changes [20]. Thus, in vulnerability assessment, the aim is not to predict precisely how a community might change in the future, as in community models, but rather to show which communities are likely to experience consequential impacts and inform future research and management efforts. Potential vulnerability of species to future global change has often been assessed [20–23], and community-level vulnerability assessment is becoming popular [24–26].

We developed a hierarchical typology of forest communities that can be used as the basis for monitoring and detecting change, as well as investigating potential future changes to forest communities. Our primary goals in this study were to identify existing empirical assemblages of tree species supported by recent forest inventory data from across the continental United States, determine which species were statistically important to those assemblages, and calculate measures of species dominance. Specifically, we sought to cluster observations from forest inventory data into a typology of tree species assemblages that can each be defined statistically by indicator species, then examine the major characteristics of those assemblages. To identify tree species assemblages, we used hierarchical clustering of tree species importance values from U.S. Department of Agriculture Forest Service Forest Inventory and Analysis (FIA) data. Once those assemblages were defined, indicator species analysis [27] was used to determine which species are statistically representative of each assemblage because of their high importance values relative to other species in the assemblage. We calculated measures of dominance for species to provide insights about the potential ecological roles of those species in existing forest tree communities [28]. Importance values and dominance measures can translate projected changes in the relative occurrence of individual species into consequential change for assemblages.

The empirical typology of assemblages and the characteristics of their species composition provide an important starting point for monitoring community changes as they occur, and assessing the potential impacts of global change drivers. While the primary aim of this paper is to present the empirical typology of species assemblages, a secondary aim is demonstrate its utility. To that end, we used the typology to explore one case-study example of how to assess the potential impacts of future climate changes on the species assemblages within a vulnerability framework. For the case study, we overlaid the clusters we defined in the eastern U.S. with climate envelope model results for individual species from Iverson et al. [29]. This case study thus used the empirical assemblages as a way to translate species-specific climate change impacts to potential climate change impacts on forest communities. In this example, we aimed not to predict the specific mix of species that may exist in the future, but rather to identify the places where projected species-level impacts of climate change may be ecologically consequential for empirical forest communities by the end of the 21^st^ century–in other words, where climate change impacts to these communities are more likely.

## Methods

### Forest inventory data

Forest plot observations from across the continental U.S. were extracted from the FIA database (FIADB version 6.0.1) [30]. The FIA program uses a sample-based statistical design to quantify forest conditions across the United States, and is the primary source for information about the status and trends of U.S. forest resources [31]. The FIA program applies a nationally-consistent sampling design of all forest and other land uses, with one permanent plot established for every 2428 ha of land [32]. FIA plots are 0.067 ha in size and consist of three 7.2-m fixed-radius subplots spaced 36.6 m apart in a triangular arrangement and one subplot of the same size in the center [30]. Data collected for forested plots by field crews include the basal area and species of every tree stem in each plot. Each FIA plot is also labeled with a forest type and forest type group via a decision tree algorithm [33]. To protect sensitive plot information, especially on privately owned lands, the publicly available FIA database contains FIA location information that has been altered slightly from the true location. We used actual plot locations to do spatial analysis (described below), but show altered locations in all figures here. To make sure we included the full set of plots for each state, we selected the set of plots that was used to produce the most recent population estimates. For most states, the most recent estimates were completed in 2013, but the most recent estimates for some states were completed in 2012, and the most recent estimate for one state (Tennessee) was completed in 2009. At each plot, we extracted the species identity and basal area of each tree greater than 2.54 cm (1 inch) diameter at breast height.

All data manipulation, and spatial and statistical analysis was done in R version 3.3.0 [34]. To avoid skewing clusters toward the most rare species, we eliminated species from the FIA data that occurred in fewer than 250 plots (0.2% of plots) [35]. We also combined some species that were varieties or were labeled with generic names, following the logic of Potter and Hargrove [36]. In some cases, as in the case of hickory species, we eliminated records with generic labels because the generic labels represented a small proportion of all records for the genus in the database. In other cases, as for hawthorn species, we combined all records into a generic record, because those species are difficult to identify in the field, and the generic label represented a large proportion of the records for all species in the genus. We excluded plots that were labeled with nonnative forest types and excluded records of nonnative species. We also excluded plots that were labeled as "nonstocked" because those indicate plots with few or no trees.

Initial inspections of the data indicated that a large number of plots (> 17,000 plots, or > 13% of the total) contained Douglas-fir (*Pseudotsuga menziesii*), and most of those would be grouped into a single cluster. Most authorities currently recognize two varieties of Douglas-fir: coast Douglas-fir (*Pseudotsuga menziesii* var. *menziesii*) and Rocky Mountain or interior Douglas-fir (*P*. *menziesii* var. *glauca*) but those are not distinguished in the FIA database. Therefore, Douglas-fir in plots occurring in California or in the western portion of the species' range west of the Cascade mountains in Washington and Oregon was labeled as coast Douglas-fir and all others were labeled as Rocky Mountain Douglas-fir, after Giunta et al. [37].

After filtering, the result was a data set of abundance and basal area for each of 176 species (see S1 Table in supplementary material for full list of species) in 127 622 plots. We calculated importance values (IV) by species and plot. The relative IV for a species is defined as the average of each species’ relative basal area and abundance in each plot, multiplied by 100. We used the IVs as the basis for clustering.

### Clustering of tree species assemblages

Multivariate statistical methods including clustering for classifying species assemblages are well developed in vegetation ecology [38–40]. Hierarchical clustering was the desired method here because species assemblages are often thought of as nested with various levels in a hierarchy. Hierarchical clustering requires a distance or dissimilarity matrix, but the size of a distance matrix for our full data set would exceed the memory limit for many contributed packages in R (for example, the vegan package has a limit of 2 GB). Therefore, we used the method of cluster seeding to reduce the number of rows in the species matrix prior to hierarchical clustering [41]. This method involves an initial step of k-means clustering, which does not require creation of a distance matrix. In the k-means clustering, a relatively large number of clusters (k) is specified in order to create cluster “seeds” that are then input to hierarchical clustering. The initial k-means clustering with a large k finds groups of plots that have identical or nearly identical information. This method was appropriate for our data because the FIA database contains many plots with identical or nearly identical species importance values, as is the case for plots that only have a single species present. For the initial k-means clustering, we used k = 20 000 clusters, and set the number of starts to 5 and the maximum iterations to 100. The results from the k-means cluster seeding were: (1) a 20 000-row x 178-column matrix in which the values were the average IV by species for each of the k-means clusters, and (2) a vector of length 127 622 indicating membership in each of the 20 000 clusters for each of the forest inventory plots.

The two results from k-means cluster seeding were used as inputs into hierarchical clustering. As our association matrix *d*, we calculated Bray-Curtis dissimilarity based on the k-means cluster matrix using the vegdist function in the vegan package in R [42]. For clustering, we used the hclust function in the R stats package [34], and specified the number of observations (cluster size) in each of the 20 000 k-means clusters, indicating that *d* is a dissimilarity matrix between existing clusters. Several hierarchical clustering linkage methods were explored. Visual inspection of the resulting dendrograms favored the 'average' linkage method, which defines clusters based on average distances among plots in pairs of clusters.

We determined the optimum number of clusters using indicator species analysis [27] because we desired empirical tree assemblages that were each identifiable based on one or more characteristic species. Indicator species are those with high specificity and fidelity to a given cluster, and are thus the most prominent members of the cluster [39]. We used the plot data set containing importance values to perform indicator species analysis for each level of the hierarchy from 2 to 200 clusters. For each of those levels, the hierarchical cluster dendrogram was cut and resulting cluster memberships were assigned back to the 127 622 plots, then indicator species analysis was run. The result of each iteration of indicator species analysis was an indicator value and p-value for each species in the data set.

Once this iterative analysis was run, the optimum number of clusters was selected based first on the criterion that all clusters in a given level had at least one significant indicator species each. Within the set of cluster typologies that met that criterion, we used several indices based on the indicator species analysis to select optimal numbers of clusters. We sought levels of the hierarchy that simultaneously maximized the sum of significant indicator values and the total number of indicator species, and minimized the average of significant p-values [27,43]. In addition to diagnostic indices from indicator species analysis, we used silhouette widths [44] to select optimal typologies within the set of typologies that had at least one indicator species per cluster. Silhouette widths compare the similarity of samples within a cluster to samples in another cluster. A high positive silhouette width indicates that a sample is more similar to clusters within its assigned cluster and thus fits well into that cluster. A low or negative silhouette width indicates greater similarity to another cluster, and therefore a poor fit. We therefore sought to choose a typology that maximized the average width, in addition to the three indicator species indices described above. We did not combine the indices quantitatively to select optimal levels of the hierarchy, but rather inspected the values of each of the indices and selected levels of the hierarchy that performed well across the indices. Indicator species analysis and silhouette widths were done in R using contributed packages cluster [45] and labdsv [46], respectively.

Within each cluster in the optimal typology, we also calculated a species dominance index (SDI) [28]for every species [28]. Frieswyk et al. [28] defined the SDI for a given species as in a community as:

$$SDI=\frac{MC+MSS+THC}{3}$$

where *MC* is the mean cover of the species across all plots and *MSS* is mean species suppression, defined as the average of the inverse of species richness in the plots in which the species occurs. *THC* is the tendency toward high cover, calculated as the number of plots in which a species meets two dominance criteria divided by the number of plots in which the species occurs. The two dominance criteria are that a species must have greater than 25% cover as well as the most cover of any species in a plot. Here, we use relative IVs (defined above) in place of cover measures. While other measurements or combinations of metrics could be used to measure dominance, we used the SDI as defined by Frieswyk et al. because it is based on three main ways in which a species can be dominant; that is, a species can have high cover in a large number of plots, can occur with few other species or can have high cover but only occur in a small number of plots [28]. In addition, the index has been used as a measure of dominance in other studies of community structure [28,47]. A cutoff value must be chosen for SDI to represent dominant species. We used a cutoff of species in 90th percentile of SDI values to represent dominant species, as used by Frieswyk et al [28]. This choice does matter, and we also explored alternative cutoffs of the 95^th^ and 85^th^ percentiles.

### Case study: Potential climate change impact for tree species assemblages in the Eastern U.S.

As one example of how the clusters and information on their community composition could be used to assess the potential impacts of change drivers on forest communities, we used projections of climate change impacts on individual tree species from the USDA Forest Service’s Climate Change Tree Atlas [29,48]. Tree Atlas data represent projected changes in IVs for individual tree species from current (1961–1990) climate and future (2070–2099; hereafter, “2100”) under a set of emissions scenarios and global climate models (GCMs). The projections under the A1FI emissions scenario according to the Hadley GCM (hereafter “Hadley High”; high emissions, relatively high projected change) and the B1 emissions scenario according to the PCM GCM (hereafter “PCM Low”; low emissions, relatively mild projected change) were used here. The spatial extent of the projected data covers the Eastern U.S. [29], and therefore our case study is restricted to that extent.

We defined potential climate change impact for a given cluster in terms of the projected amount of change in IV for all of the cluster’s dominant species that were previously identified, using the same 90^th^ percentile cutoff for SDI described above. For each plot location, we extracted the modeled change in IV under each of the two emissions scenarios from the Tree Atlas spatial projections for all dominant species in the cluster to which that plot was assigned. As our measure of overall impacts to the tree species assemblage for each cluster, we calculated the mean change in dominant species IV across all plots in a cluster, weighted by each dominant species’ SDI value. To examine spatial patterns of impact within each cluster, we calculated the mean change in IV at each plot location across all dominant species in the assemblage to which that plot was assigned.

When summarizing changes in IV across assemblages, it is important to note that the reliability of Tree Atlas projections varies by species [29]. Here, we restricted our analysis to clusters: (1) that contained at least 100 plots, (2) for which all dominant species were included in the Tree Atlas projections, and (3) for which Tree Atlas projections for the majority of dominant species had at least medium reliability.

## Results

### Clustering of tree species assemblages

**Fig 1:**
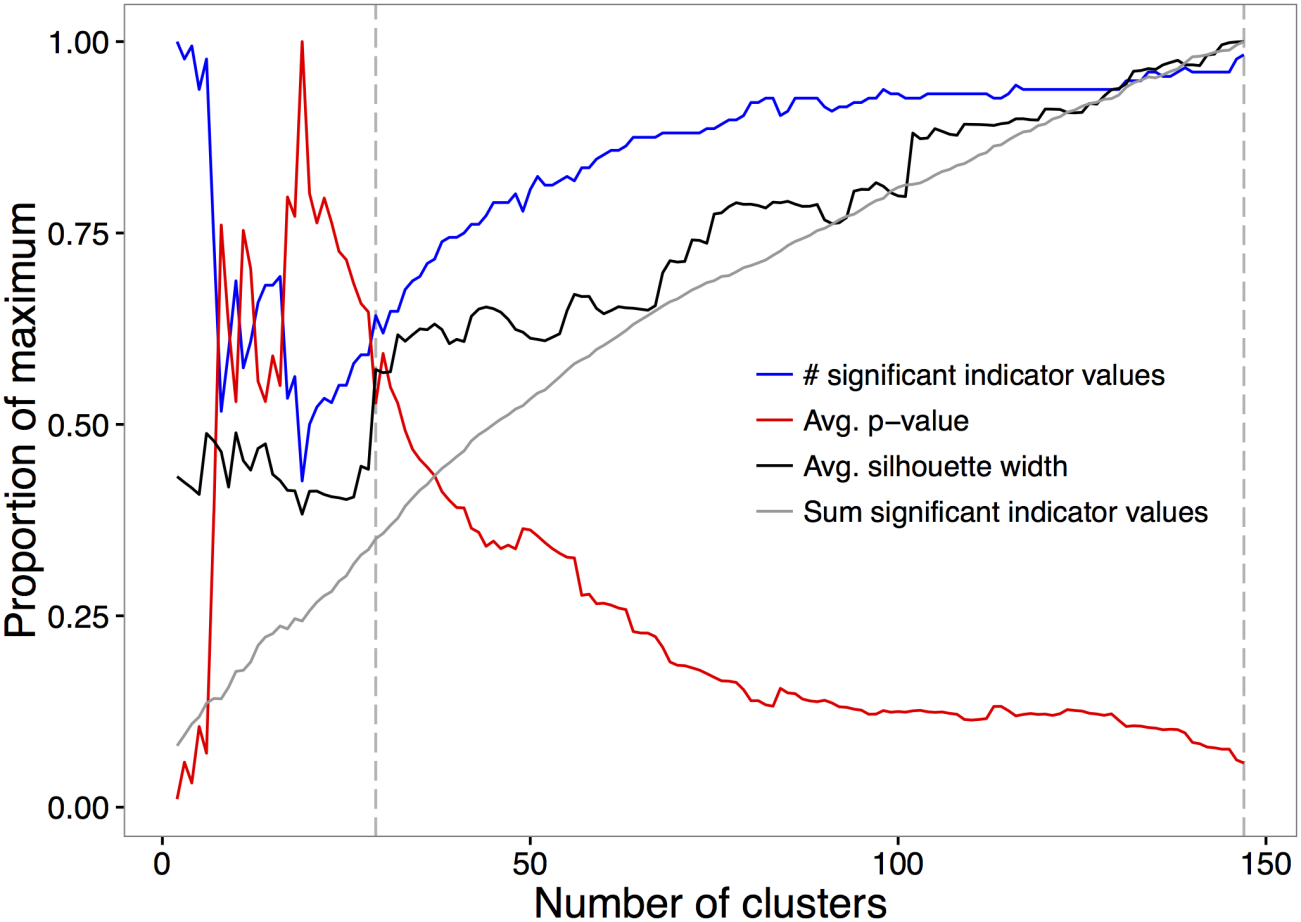
Diagnostic indices used to select groupings in the hierarchical clustering. Dashed vertical lines correspond to selected typologies of 29 (left) and 147 (right) cluster.

Indicator species analysis of hierarchical clustering showed that cluster typologies with 2 to 147 clusters had at least one indicator species per cluster. Within that range, 147 clusters maximized the sum of significant indicator values, the total number of indicator values, and the average silhouette width, while minimizing the average p-values (Fig 1). For k = 147, the sum of significant indicator values was 101.1, the total number of indicator species was 173, the average of the p-values was 0.006, and the average silhouette width was 0.182. In addition, a grouping of 29 tree species assemblages resulted in substantially larger values for the total number of indicator values and the average silhouette widths compared to groupings with slightly smaller or larger numbers of clusters; in other words, local maxima for those diagnostic criteria. Average p-values showed a local minimum at 29 groupings also (Fig 1). For k = 29, the sum of significant indicator values was 35.5, the total number of indicator species was 111, the average of the p-values was 0.050, and the average silhouette width was 0.104. Thus, we further investigated both the 29- and 147-cluster typologies (Fig 1).

The largest clusters in the 29-cluster typology corresponded to over 20 groups in the nested 147-cluster typology, while the membership of plots in twelve clusters remained unchanged in both levels of the hierarchy (Table 1, S2 Table and S1 Fig in supplementary material). Sizes of clusters varied widely within both typologies. Groups in the 29-cluster typology contained between 6 and 30 068 plots (< 0.01% and 23.6% of all plots, respectively; Table 1), while groups in the 147-cluster typology included between 4 and 12 796 plots (< 0.01% and 10.0% of all plots; S2 Table).

**Table 1:** Significant indicator species, dominant species, and average characteristics of the 29 broad assemblages, in order corresponding to Fig 2. Species in bold have the highest indicator values and are the ones by which we refer to each assemblage.

Indicator species	Ind. Val.	p-value	Num. plots	Num. specific assemblages	Median richness (species/plot)	Mean basal area (m^2^/ha)	Mean abundance (trees/plot)	Dominant species	SDI
**slash pine**	0.72	0.001	2934	4	2	21.40	25.98	slash pine	0.58
**longleaf pine**	0.31	0.001						longleaf pine	0.32
pondcypress	0.13	0.006						pondcypress	0.22
turkey oak	0.08	0.02						turkey oak	0.13
sweetbay*	0.06	0.043							
**balsam fir**	0.41	0.001	12613	11	4	27.16	35.27	quaking aspen	0.32
**quaking aspen**	0.32	0.001						red pine	0.24
paper birch*	0.27	0.001						northern white cedar	0.18
black spruce	0.19	0.001						balsam fir	0.17
northern white cedar	0.19	0.002						black spruce	0.16
white spruce	0.15	0.004						jack pine	0.16
black ash	0.15	0.005						tamarack (native)	0.13
red spruce	0.14	0.005						black ash	0.12
tamarack (native)	0.14	0.008						Jeffrey pine*	0.11
red pine	0.12	0.009						red spruce	0.10
yellow birch*	0.09	0.017							
jack pine	0.07	0.041							
**common persimmon**	0.98	0.001	83	1	2	9.17	7.08	common persimmon	0.71
								black cherry*	0.11
**butternut**	1.00	0.001	6	1	2	11.99	4.50	butternut	0.81
sweet birch*	0.11	0.011							
**sourwood**	0.98	0.001	6	1	3	14.17	8.83	sourwood	0.74
scarlet oak*	0.28	0.001							
Virginia pine*	0.10	0.007							
**sugar maple**	0.27	0.001	30068	24	7	29.29	27.62	sugar maple	0.17
**red maple**	0.23	0.001						red maple	0.17
American beech*	0.20	0.001						eastern white pine	0.12
white oak	0.18	0.001						white oak	0.12
northern red oak*	0.22	0.002						chestnut oak	0.11
yellow poplar	0.13	0.004						yellow poplar	0.11
eastern hemlock	0.11	0.007						eastern hemlock	0.11
chestnut oak	0.12	0.009						Virginia pine*	0.10
eastern white pine	0.08	0.02							
black oak*	0.09	0.021							
pignut hickory*	0.08	0.021							
mockernut hickory*	0.06	0.048							
**loblolly pine**	0.48	0.001	25575	21	6	28.57	29.67	loblolly pine	0.40
**sweetgum**	0.20	0.001						water tupelo*	0.15
water oak*	0.13	0.003						pond pine*	0.14
shortleaf pine	0.12	0.009						sweetgum	0.13
post oak	0.11	0.016						post oak	0.12
southern red oak*	0.10	0.017						eastern redcedar	0.12
eastern redcedar	0.08	0.026						baldcypress*	0.12
blackgum*	0.06	0.032						shortleaf pine	0.10
winged elm*	0.07	0.034						swamp tupelo*	0.10
American holly*	0.06	0.039						loblolly bay*	0.10
**hawthorn spp.**	0.97	0.001	88	1	2	9.06	11.10	hawthorn spp.	0.65
**American plum**	0.03	0.05						eastern hophornbeam*	0.18
								black willow*	0.18
								blackgum*	0.17
								American elm*	0.13
								shagbark hickory*	0.13
								American plum	0.12
								serviceberry spp.*	0.12
**black willow**	0.92	0.001	343	1	3	29.43	15.37	black willow	0.65
								green ash*	0.13
								eastern redbud*	0.11
**green ash**	0.14	0.005	9303	32	5	23.36	17.53	silver maple	0.19
**American elm**	0.12	0.005						northern pin oak*	0.19
black walnut	0.13	0.007						green ash	0.17
hackberry	0.13	0.009						eastern cottonwood*	0.16
bur oak	0.09	0.011						bur oak	0.16
slippery elm*	0.07	0.031						black locust	0.15
bitternut hickory*	0.07	0.036						boxelder	0.13
boxelder	0.07	0.042						sugarberry	0.12
black locust	0.07	0.046						Osage orange*	0.12
American sycamore	0.06	0.047						American elm	0.11
silver maple*	0.05	0.05						hackberry	0.11
								American sycamore	0.11
								shagbark hickory*	0.11
								pecan*	0.10
								pin oak*	0.10
								black walnut	0.10
								overcup oak*	0.10
**velvet mesquite**	1.00	0.001	269	1	1	6.43	7.58	velvet mesquite	0.97
								redberry juniper*	0.10
**chittamwood**	0.98	0.001	18	1	1	4.47	4.67	chittamwood	0.90
**honey mesquite**	0.78	0.001	3761	3	1	6.15	8.40	honey mesquite	0.81
**Pinchot juniper**	0.12	0.003						Pinchot juniper	0.34
redberry juniper	0.05	0.035						redberry juniper	0.32
								oneseed juniper*	0.17
**cedar elm**	0.97	0.001	149	1	3	15.48	15.70	cedar elm	0.64
								willow oak*	0.20
								water oak*	0.15
								Osage orange*	0.15
								eastern redcedar*	0.11
**live oak**	0.52	0.001	1790	4	2	16.80	18.57	Ashe juniper	0.53
**Ashe juniper**	0.52	0.001						live oak	0.43
Texas persimmon	0.10	0.022						cabbage palmetto	0.29
cabbage palmetto	0.10	0.03						Texas persimmon	0.11
**California live oak**	0.85	0.001	224	2	1	21.72	13.46	California live oak	0.73
**California laurel**	0.29	0.001						California laurel	0.32
								blue oak*	0.13
								red alder*	0.12
**blue oak**	0.68	0.001	585	3	1	15.26	12.60	blue oak	0.65
**interior live oak**	0.35	0.001						interior live oak	0.38
California foothill pine	0.23	0.001						Pacific dogwood*	0.33
								California foothill pine	0.20
								singleleaf pinyon*	0.14
**Gambel oak**	0.92	0.001	769	1	2	15.31	26.65	Gambel oak	0.82
**alligator juniper**	0.79	0.001	736	2	3	18.05	18.09	alligator juniper	0.41
**Arizona white oak**	0.52	0.001						Arizona white oak	0.31
Emory oak	0.36	0.001						Emory oak	0.21
								ponderosa pine*	0.14
**Utah juniper**	0.59	0.001	7789	3	2	19.44	17.70	Utah juniper	0.49
**two needle pinyon**	0.36	0.001						oneseed juniper	0.35
oneseed juniper	0.22	0.002						singleleaf pinyon	0.29
singleleaf pinyon	0.20	0.001						two needle pinyon	0.28
**black cottonwood**	0.99	0.001	44	1	1	36.11	8.61	black cottonwood	0.86
**bigleaf maple**	0.07	0.038						bigleaf maple	0.20
**western juniper**	0.73	0.001	1168	2	1	15.20	13.94	western juniper	0.69
**curlleaf mountain mahogany**	0.31	0.001						curlleaf mountain mahogany	0.39
								ponderosa pine*	0.17
**lodgepole pine**	0.50	0.001	6069	4	2	27.71	34.54	lodgepole pine	0.46
**subalpine fir**	0.48	0.001						subalpine fir	0.31
Engelmann spruce	0.47	0.001						Engelmann spruce	0.26
whitebark pine	0.13	0.005						whitebark pine	0.14
**Rocky Mountain Douglas-fir**	0.45	0.001	11585	7	2	25.30	22.89	ponderosa pine	0.46
**ponderosa pine**	0.30	0.001						Rocky Mountain Douglas-fir	0.36
grand fir	0.19	0.002						grand fir	0.20
western larch*	0.12	0.005						Rocky Mountain juniper*	0.17
								western redcedar*	0.14
**chokecherry**	0.99	0.001	13	1	3	13.86	11.23	chokecherry	0.75
Pacific dogwood*	0.06	0.02							
**Oregon white oak**	0.97	0.001	213	1	2	20.54	20.92	Oregon white oak	0.74
								ponderosa pine*	0.10
**canyon live oak**	0.71	0.001	409	2	3	28.29	28.21	canyon live oak	0.57
**California black oak**	0.42	0.001						California black oak	0.36
**mountain hemlock**	0.74	0.001	919	2	3	50.49	41.00	Pacific silver fir	0.42
**Pacific silver fir**	0.64	0.001						mountain hemlock	0.41
noble fir	0.17	0.001						noble fir	0.19
western white pine*	0.06	0.046						Pacific yew*	0.11
**coast Douglas-fir**	0.54	0.001	10093	9	3	49.83	34.59	coast Douglas-fir	0.45
**western hemlock**	0.23	0.001						white fir	0.22
white fir	0.15	0.005						redwood*	0.20
red alder	0.10	0.013						California red fir*	0.18
incense cedar*	0.08	0.021						Jeffrey pine*	0.18
western redcedar*	0.07	0.032						western hemlock	0.17
tanoak	0.07	0.039						tanoak	0.13
giant chinkapin*	0.04	0.04						red alder	0.10

*This species is either an indicator for the corresponding assemblage but is not dominant, or is dominant but not an indicator.

Groups in the 29-cluster typology had between one and twelve indicator species, and between one and seventeen dominant species each (Table 1, see S3 Table for a comparison of results from the two alternative cutoff values for dominant species), while groups in the 147-cluster typology had between one and three indicator species each (S2 Table). The 90^th^ percentile value of SDI, which was used to determine whether species were designated as “dominant” in the 29-cluster typology was 0.096. The alternative 85^th^ and 95^th^ percentile values were 0.060 and 0.168, respectively. The range of indicator values that were significant for each group, especially in the 29-cluster typology, demonstrates that the specificity and fidelity of indicator species varied across those groups (Table 1). With fewer indicator and dominant species per cluster, the 147-cluster grouping represents specific tree species assemblages that are each represented by a small number of species (hereafter, “specific assemblages”), while the 29-cluster grouping represents generally broader assemblages (hereafter, “broad assemblages”), although as stated above, some groups did not vary between the two typologies. Because of the large number of specific assemblages identified, we focus on results and summaries for the broad assemblages, and examine specific assemblages that correspond to some of them. Hereafter, we refer to broad and specific assemblages that have more than one indicator species using the common names of the two most significant indicator species, and refer to those with a single indicator species using the name of that species (Table 1).

**Fig 2:**
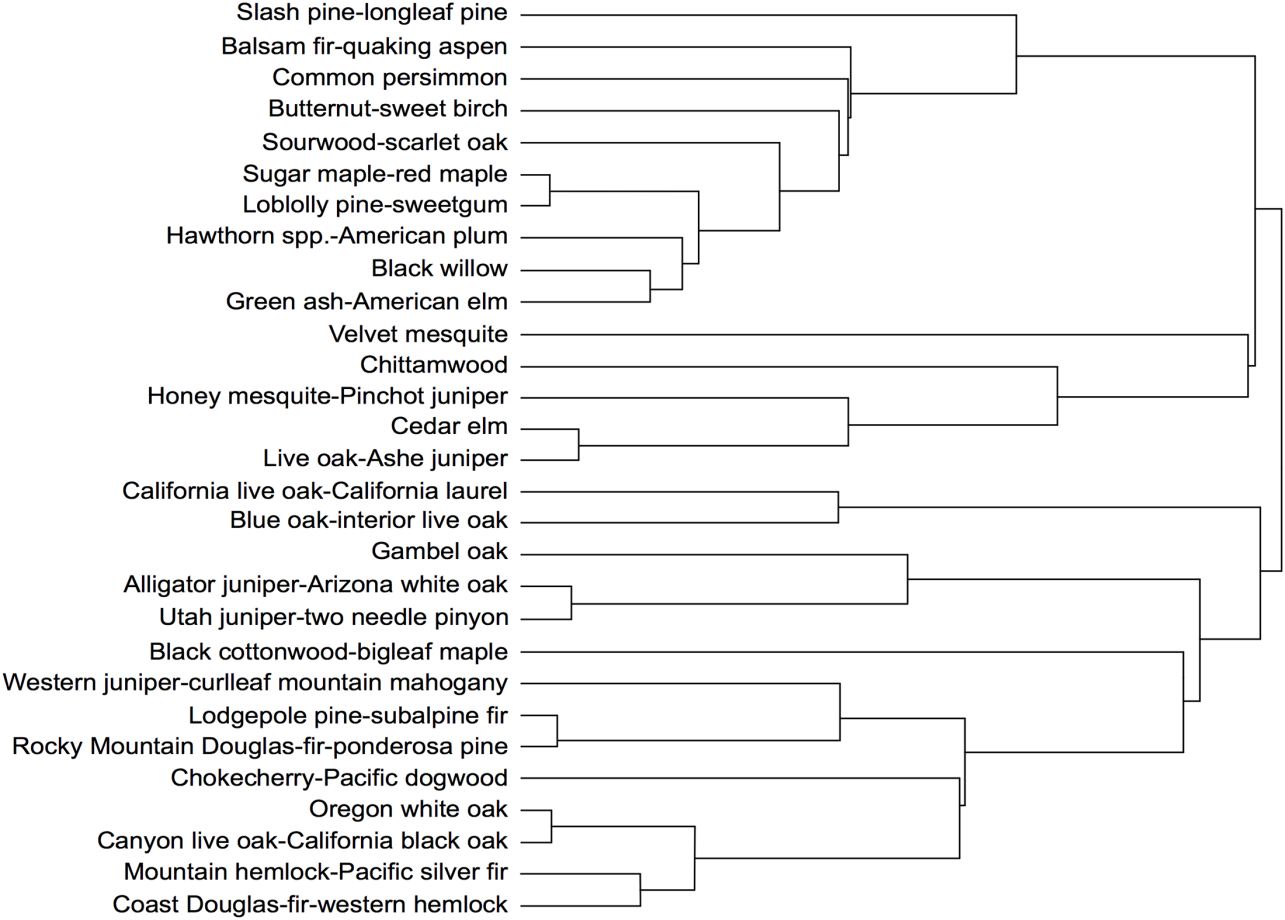
Dendrogram from hierarchical clustering of tree species importance values. The 29 broad assemblages are shown. See S1 Fig for dendrogram cut at 147 specific assemblages.

**Fig 3:**
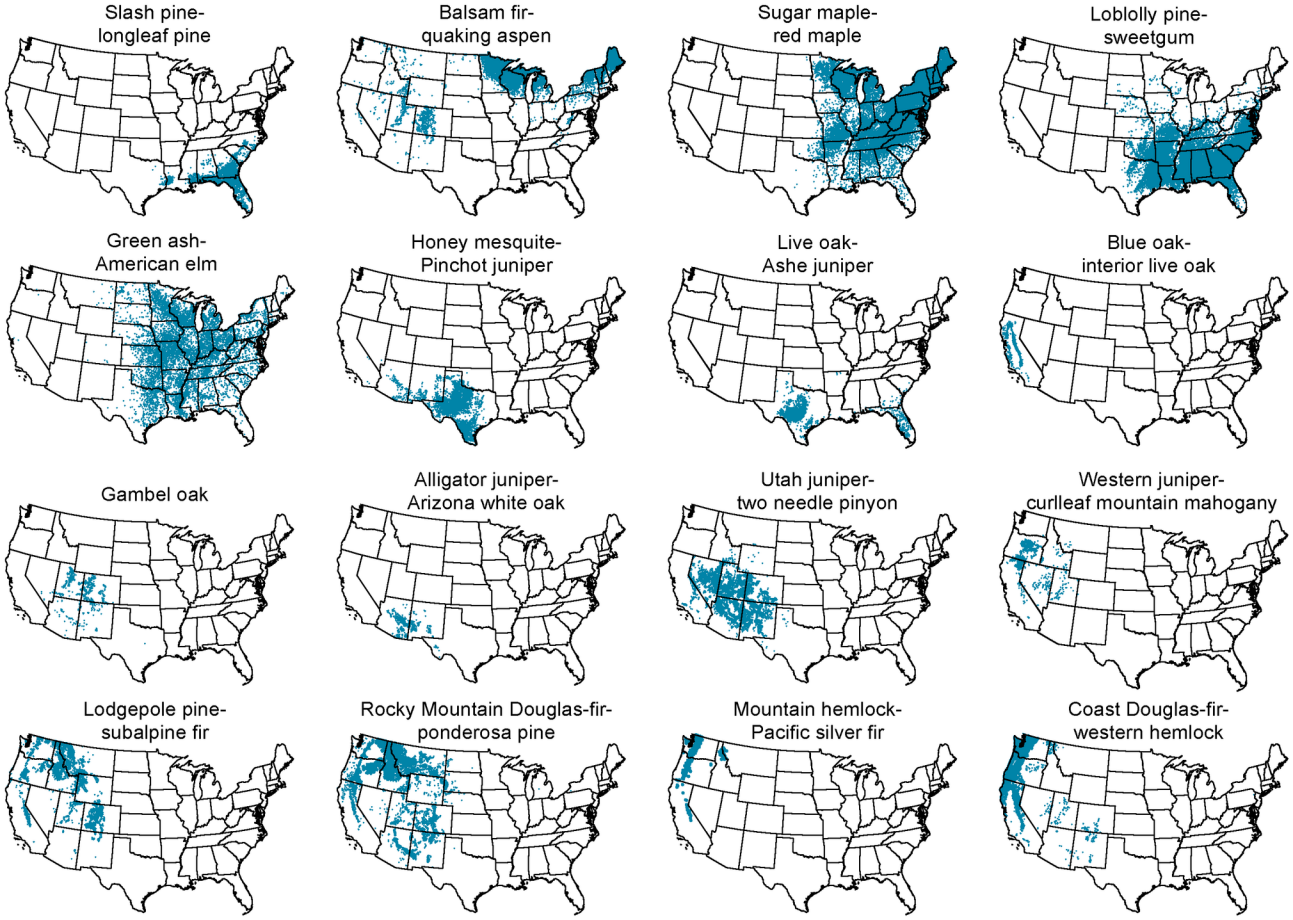
Locations of FIA plots in the 29 broad assemblages. The sixteen assemblages containing more than 500 plots are shown. When read left to right along each row from top to bottom, the order of the assemblages corresponds to their order from top to bottom in the dendrogram (Fig 2).

The broad assemblages were grouped in the dendrogram generally by their geographic extents, with assemblages at the top of the dendrogram occurring largely in the eastern U.S., those in the middle occurring in the central U.S. and Texas, and those at the bottom in the western U.S. (Fig 2). In other words, the rightmost splits in the dendrogram differentiated assemblages occurring in the eastern versus the central versus the western U.S. (Fig 2). Some were distributed over wide ranges of longitude (e.g. the balsam fir-quaking aspen assemblage), latitude (e.g. the western juniper-curlleaf mountain mahogany assemblage), or both (e.g. the sugar maple-red maple and green ash-American elm assemblages; Fig 3). Most broad assemblages tended to be distributed in either the eastern or the western U.S. only, though there were exceptions, including the balsam fir-quaking aspen assemblage (Fig 3).

**Fig 4:**
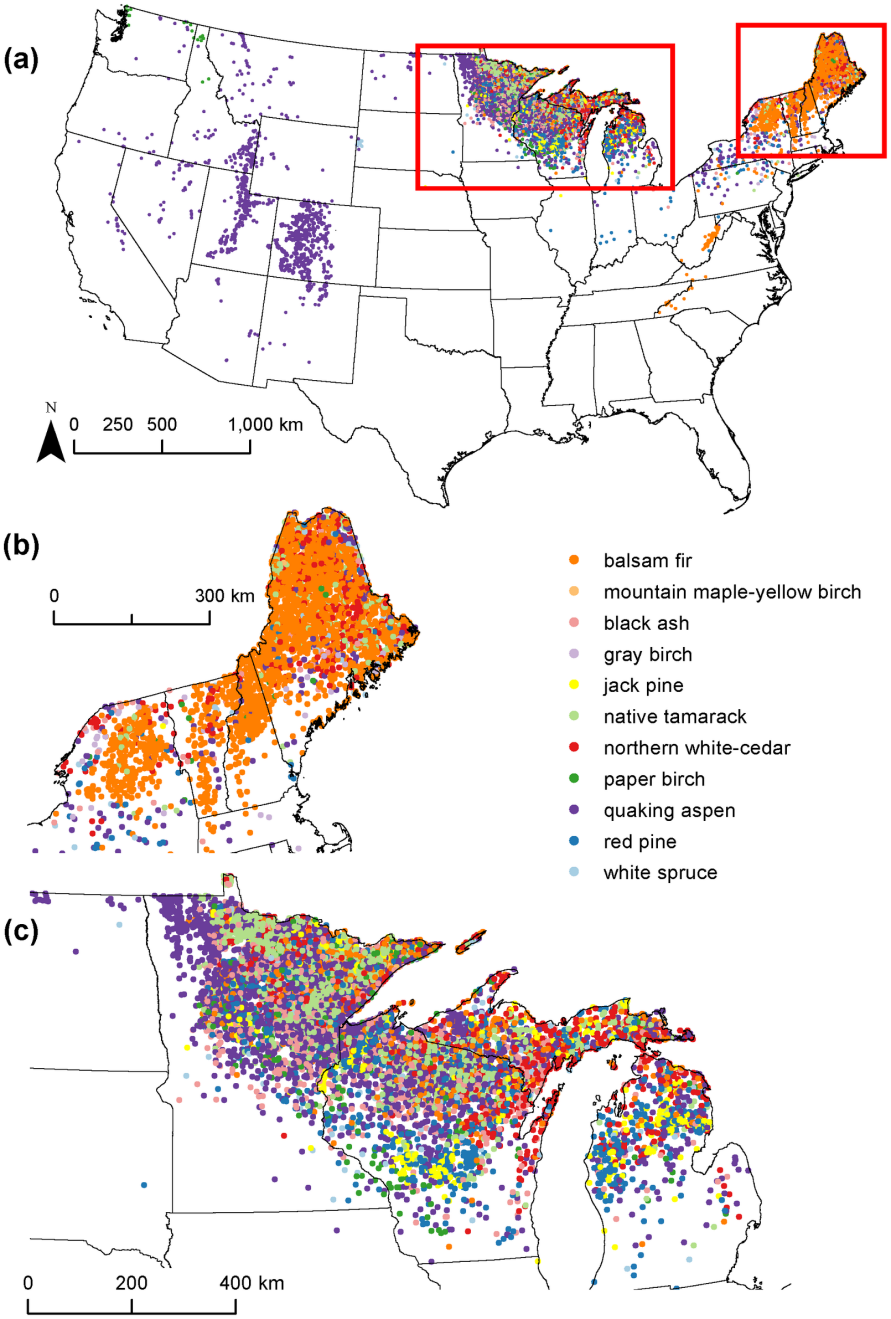
Forest inventory plots in the balsam fir-quaking aspen broad assemblage. Symbol colors represent each plot’s membership in a specific assemblage. (A) all plots; (B) plots in New England; (C) plots in the western Great Lakes region.

The broad assemblages varied in their community characteristics. We next summarize some of the major characteristics for a few example assemblages, including the significant indicator species, relative size in terms of number of plots, as well as average plot-level species richness, basal area and tree abundance (Table 1). First, the balsam fir-quaking aspen assemblage is an example that represented widespread a widely-distributed community with high plot-level tree species richness on average, and a large number of indicator species. The assemblage was distributed on plots in the Northeast and upper Midwest, as well as where quaking aspen occurs in the West (Fig 4a). In addition to balsam fir and quaking aspen, there were ten other significant indicator species for the assemblage (Table 1). Most of the indicator species are also dominant, except for paper birch and yellow birch. The broad assemblage corresponded to eleven specific assemblages having 14 indicator species altogether. The quaking aspen assemblage was the dominant specific assemblage in the western portion (Fig 4a), while in New England, a mix of specific assemblages occurred, but the balsam fir assemblage was most common there (Fig 4b). A mix of specific assemblages also occurred in the upper Midwest (Fig 4c). Like the balsam fir-quaking aspen assemblage, the green ash-American elm, loblolly pine-sweetgum, and sugar maple-red maple assemblages were also widespread with a large number of significant indicator species. Each of these assemblages had higher average plot-level tree richness than the balsam fir-quaking aspen assemblage, but lower plot-level average tree abundances.

**Fig 5:**
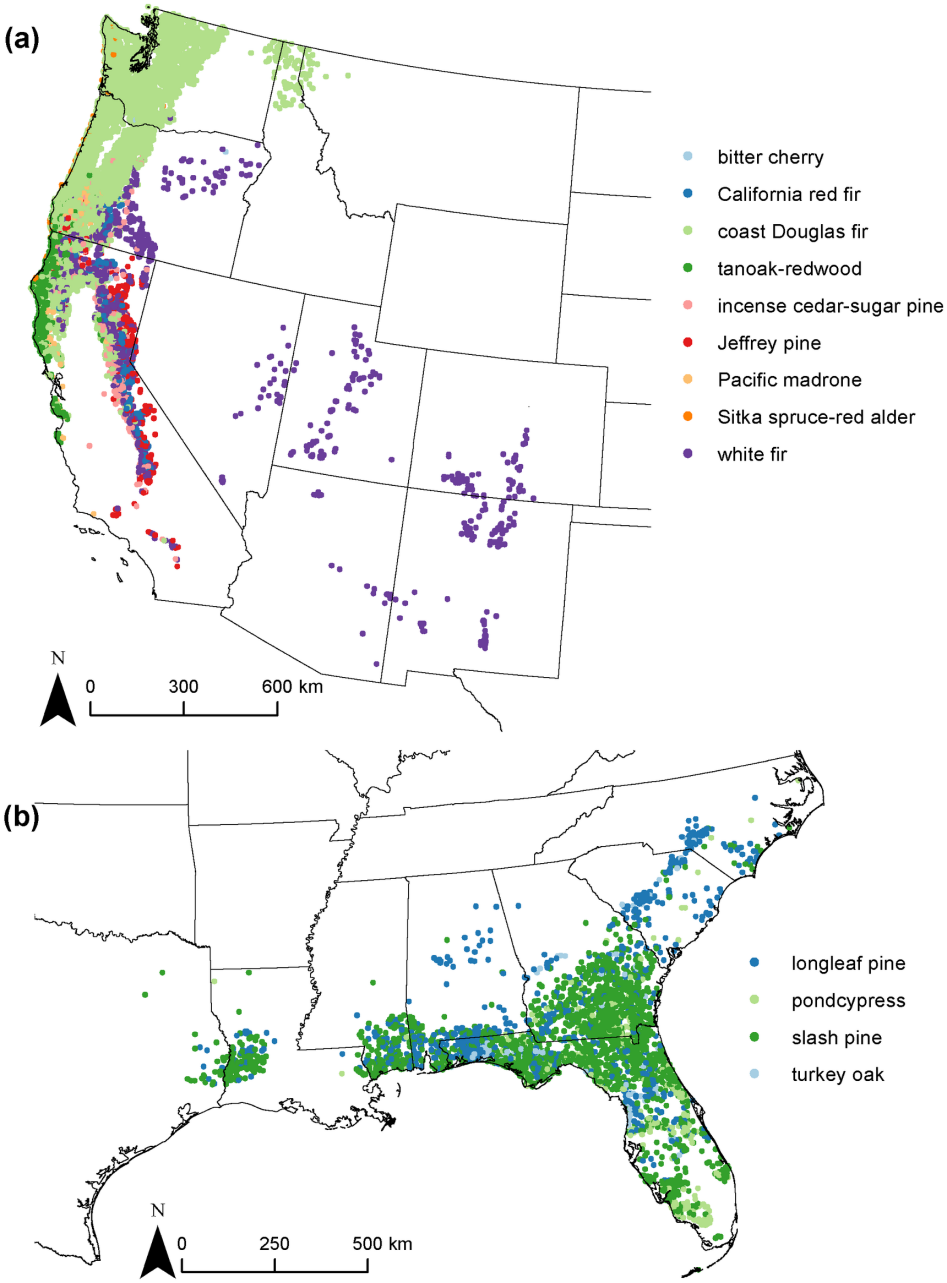
Forest inventory plots symbolized according to their membership in specific assemblages. (A) plots in the coast Douglas-fir-Western hemlock broad assemblage; (B) plots in the slash pine-longleaf pine broad assemblage.

Second, the coast Douglas-fir-western hemlock assemblage was an example of an assemblage with lower average plot-level species richness, but high plot-level basal areas and tree abundances. The substantially higher indicator value for coast Douglas-fir (i.e., approximately two times larger or more) compared with the other significant indicator species suggests that the assemblage was characterized best by coast Douglas-fir. The species also has a higher SDI value compared with other dominant species in the assemblage, showing that it is dominant most often. The assemblage included plots in coastal portions of California, Washington and Oregon, as well as in the forests of the Sierra Nevada mountains, and where white fir occurs to the east (Fig 5a). In addition to coast Douglas-fir and western hemlock, six other species were indicators of the broad assemblage (Table 1). Nine specific assemblages corresponded to this broad assemblage (Fig 5a). Most plots, especially in Oregon, Washington and Northern California, belonged to the assemblage with coast Douglas-fir as the single indicator species. The assemblage with giant chinkapin, redwood, and tanoak as indicator species occurred in coastal California, while the two assemblages with Jeffrey pine and white fir as indicators were present in the Sierra Nevada Mountains.

Several other assemblages had some similar characteristics to the coast Douglas-fir-western hemlock assemblage, including lodgepole pine-subalpine fir and mountain hemlock-Pacific silver fir. In contrast to the coast Douglas-fir-western hemlock assemblage, the lodgepole pine-subalpine fir assemblage had moderate basal area, and high indicator values for three tree species instead of just one.

The third example, the slash pine-longleaf pine broad assemblage, was most characterized by a single indicator species (slash pine) that had a higher indicator value than the other indicator species, but the assemblage had relatively low average plot-level tree species richness, and moderate average plot-level basal area and tree abundance. That assemblage occurred on plots in the southeastern U.S. from eastern Texas to North Carolina. In addition to slash pine and longleaf pine, pondcypress, turkey oak, and sweetbay were indicator species of the broad assemblage, and the four indicator species were also the dominant species. Four specific assemblages corresponded to the broad assemblage, with the slash pine assemblage being the largest and dominating throughout, except in North and South Carolina, where the longleaf pine assemblage was present (Fig 5b).

### Case study: Assessment of potential climate change impacts across assemblages

**Fig 6:**
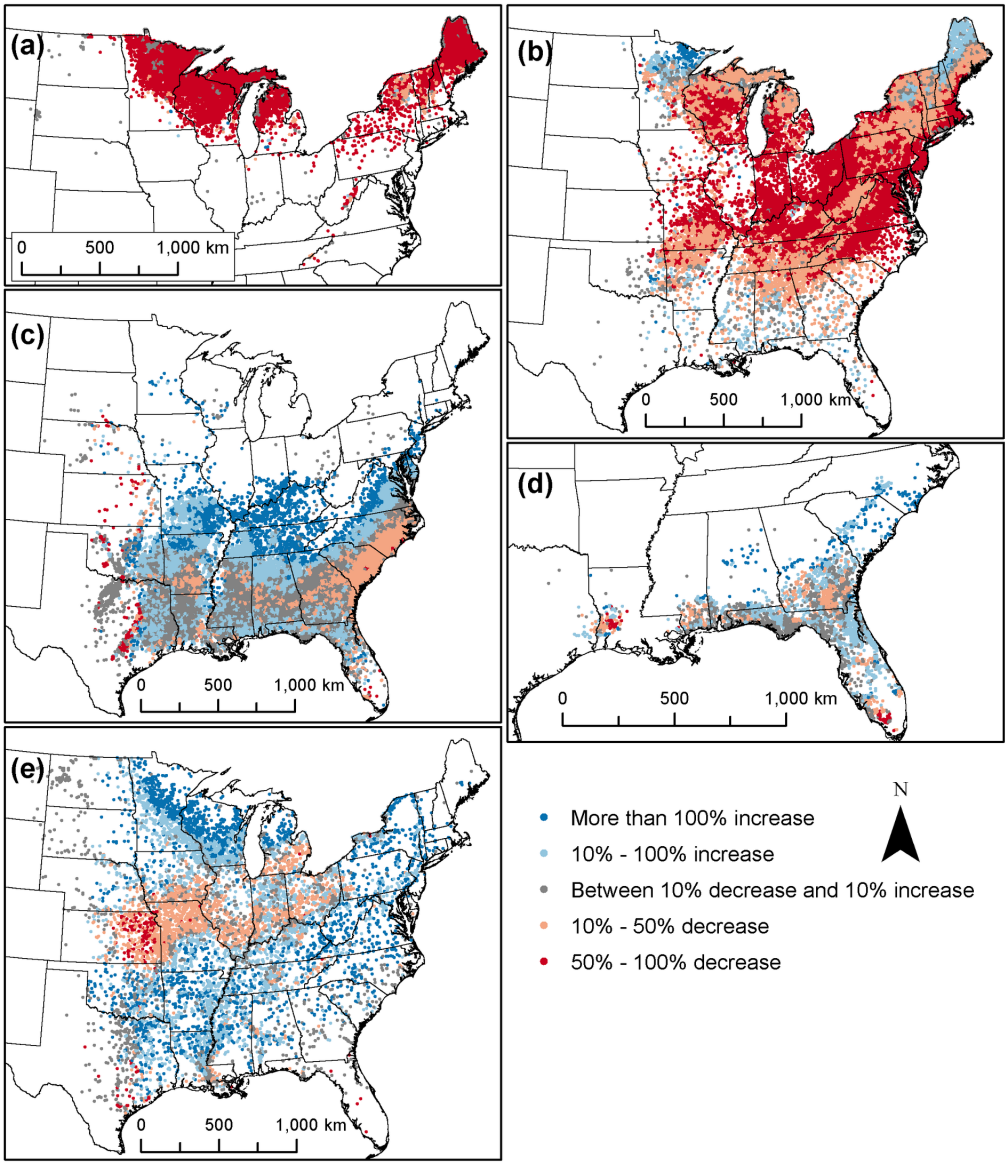
Projected change in importance value for dominant species in five broad assemblages. Maps show projected changes under the Hadley High scenario for plots in the (A) balsam fir-quaking aspen assemblage which had the largest projected decrease in importance value on average, (B) sugar maple-red maple assemblage, (C) loblolly pine-sweetgum assemblage, (D) slash pine-longleaf pine assemblage, and (E) green ash-American elm assemblage, which had the largest projected increase in importance value on average.

The criteria we used for exclusion of broad assemblages from the climate change impact assessment resulted in five of the 29 retained: balsam fir-quaking aspen, green ash-American elm, loblolly pine-sweetgum, slash pine-longleaf pine, and sugar maple-red maple (Table 2). These five assemblages together contained 79 342 plots (94.3% of the 84 177 plots in the eastern U.S., and 62.2% of the 127 622 total plots). Average projected changes in IV for dominant species within those broad assemblages ranged from a decrease for some broad assemblages to an increase for others under each scenario (Table 2). Projections under the Hadley High scenario spanned a larger range of values, including greater increases and greater decreases in average IV projected for assemblages under Hadley High compared with PCM Low. The broad assemblages also varied in the geographic patterns of their projected changes in IV (Fig 6 shows geographic results under the Hadley High scenario; results for PCM Low are in S2 Fig in supplementary material).

**Table 2:** Potential climate change impacts to five broad assemblages under two climate change scenarios. Here, impact is defined as the average change in importance value for dominant tree species projected across all plots in the assemblage by the end of the 21^st^ century.

Broad assemblage	Num. plots in east	Prop. of plots^a^	PCM Low % change	Hadley High % change
balsam fir-quaking aspen	11486	91%	-45.4%	-74.5%
sugar maple-red maple	30068	100%	-13.0%	-48.2%
loblolly pine-sweetgum	25574	100%	7.9%	1.6%
slash pine-longleaf pine	2933	100%	13.1%	3.3%
green ash-American elm	9281	100%	26.9%	19.3%

^*a*^ The proportion of the total number of plots in the given assemblage that were included in climate change impact assessment.

The largest projected decrease in IV under both scenarios was for plots in the balsam fir-quaking aspen assemblage (Table 2). The IV for dominant species in that assemblage was projected to decrease at almost every plot location, with only a small number of plots showing an increase under Hadley High (Fig 6a). A smaller average decrease in IV was projected for the sugar maple-red maple assemblage, which was distributed across the eastern U.S. (Table 2). Throughout most of the extent of that assemblage, the average IV for dominant species was projected to decrease, but increased IV was projected for some plots, particularly the most northern and some southern plots (Fig 6b).

Small to moderate increases in IV on average were projected under both scenarios for the loblolly pine-sweetgum, slash pine-longleaf pine, and green ash-American elm assemblages (Table 2). For the loblolly pine-sweetgum assemblage, especially in the northern part of its distribution, a large proportion of plots were projected to see a substantial increase in IV for dominant species (Fig 6c). For the slash pine-longleaf pine assemblage, small regions with projected increases or decreases in IV were present throughout the distribution (Fig 6d). For the green ash-American elm assemblage, the dominant species were projected to experience moderate to large increases in IV at the majority of plot locations, except for the middle portion of the analysis extent, from Nebraska east to Michigan and Ohio (Fig 6e).

## Discussion

A consistent look at how tree species group into forest communities is essential for monitoring, assessing, and projecting future impacts to those communities from drivers such as climate change and land use change. We developed an empirical, hierarchical typology of tree species assemblages using recent forest inventory data across the continental U.S. that can be used as a baseline to assess future changes in species composition and to find places where forest communities may be most affected by these global change drivers. The typology identified two levels of a hierarchy of forest communities, and identified 111 and 173 species as indicators of those two levels, respectively.

Our empirical typology of forest inventory data for informing studies of global change impacts revealed new insights into tree species assemblages over existing national forest community classification schemes that were developed for different purposes. While there are some broad qualitative similarities with other classifications, there are important differences. In particular, some of the most widespread groups in the empirical typology do not have exact analogs in other classifications. For example, the balsam fir-quaking aspen assemblage, which is wide-ranging and occurs in the eastern and western U.S., is broader than any class in the newly-released U.S. National Vegetation Classification (USNVC) and has dominant and indicator species that occur in five forest type groups used by the USDA Forest Service. Though the assemblage shares most of its indicator species with both the Laurentian-Acadian Mesic Hardwood-Conifer Forest and Laurentian-Acadian Pine—Hardwood Forest & Woodland macrogroups in the USNVC [49,50], those macrogroups are limited to the eastern U.S., and do not include the locations in the western U.S. where quaking aspen is found. Because the USNVC was developed as a consistent standard to support conservation and management [51], most macrogroups are characterized by a single ecoregion, limiting their correspondence with wide-ranging assemblages in our typology. In addition, USDA Forest Service forest type groups were initially developed to define timber resources [52], and many tend to group species with a common genus that share a similar geographic range. More fully examining and quantifying the relative correspondence among these classification schemes would shed light on their differences and similarities, provide more insight into their relative strengths, and inform recommendations about the best uses of each. Finally, because these classification schemes were developed for different purposes, picking and choosing some classes from each could also be useful for informing management and conservation in some cases, and further work to explore that possibility is warrented.

The nested, hierarchical structure of the typology has potential for informing a wide range of ecological assessments of forest change across the U.S. In particular, the emerging field of macrosystems ecology focuses on understanding ecological processes and patterns at broad extents, while emphasizing hierarchies, multiple scales, and cross-scale interactions [53–55]. This typology would thus inform a wide range of studies of forest communities in a macrosystems framework. Specifically, the relationships between clusters in a single level of the hierarchy can be used to provide insights into observed patterns. For example, in the broad assemblages of the 29-cluster typology, the sugar maple-red maple and loblolly pine-sweetgum assemblages have similar species composition, as demonstrated by their branching toward the left of the dendrogram (Fig 2). An examination of the climate, soil, and other abiotic factors that determine their spatial distribution would shed light on whether they also occur in relatively similar environments on average. Conversely, branches for the two broad assemblages that have varieties of Douglas-fir as indicator speices, Rocky Mountain Douglas-fir-ponderosa pine and coast Douglas-fir-western hemlock, are relatively far apart on the dendrogram and their corresponding assemblages in the 147-cluster typology have different sets of indicator species. That suggests that in addition to their different geographic distributions, the two varieties also tend to associate with different sets of species. While we focused on two levels of the hierarchy here, any level of the hierarchy can be used, according to the goals of a given study, or multiple levels of the hierarchy can be used simultaneously. Further analysis at multiple levels of the hierarchy, using ancillary environmental predictor variables can shed more light on the relationships among forest communities at multiple scales.

The case-study assessment of potential climate change impacts for five broad assemblages demonstrates one method by which the empirical typology can be used for assessments of future change at broad extents. Those results point to the overall tree communities, as well as the locations within those communities, where dominant tree species may be most affected by climate change, and thus where follow-up studies and monitoring could be beneficial. The assessment indicates varying levels of overall impact to forest communities, and varying geographic patterns of those impacts, underscoring the idea that the impacts from climate change are likely to vary with composition of species and the responses of those species to climate change. The balsam fir-quaking aspen assemblage had the largest decrease in importance value (IV) for its dominant species overall, and a decrease in IV across nearly all plot locations in the study area. Indeed, recent research by Zolkos et al. [56] provides evidence across emissions scenarios and species distribution model sources that the largest losses of habitat for all eastern U.S. tree species may be three of the dominant species of this assemblage: balsam fir, quaking aspen, and red spruce. While some dominant species in the balsam fir-quaking aspen assemblage such as jack pine may have increased habitat IV in some places, projected decreases in IVs are large for most species across the assemblage’s extent [48]. In contrast, dominant species in the green ash-American elm assemblage, which is relatively evenly distributed across the eastern U.S., were projected to experience moderate increases in IV on average. Part of the reason for this increase may be that the assemblage is characterized by several dominant species with relatively low current IVs on average, and thus there is room for increases in importance for those species as climate changes.

The case-study assessment of potential climate change impacts for five broad empirical tree assemblages identified the assemblages and places for which climate change impacts are more likely by the end of the 21^st^ century. A critical next step for analysis would be to further examine the detailed population- and community-level processes in places where one or more assemblages have dominant species that are projected to experience substantial decreases in IV. An example of such analysis that already exists is the modeling done by Brandt et al. [26] for sugar maple in the Central Hardwoods region. Our assessment shows the sugar maple-red maple assemblage may experience decreases in IV in that region. Results from Brandt et al. [26] showed future establishment probabilities for sugar maple close to 0 and substantial projected declines in basal area and trees per acre by the end of the century.

In addition, the climate change impact assessment tells only part of the story about vulnerability to climate change, and should be interpreted in light of adaptive capacity. In some cases, a tree community’s dominant species may have high potential climate change impact in terms of sensitivity and exposure to climate change, but may also have high adaptive capacity, reducing their vulnerability. For example, there is evidence that occurrences of red maple have increased since the mid-20^th^ century across the east due to fire suppression and associated increasingly mesophytic conditions, and may not be as dependent on climate conditions to persist [2,26,57]. Thus the sugar maple-red maple assemblage may be becoming more common on the landscape, and may be less vulnerable to climate change than our assessment of changes in IV indicates. On the other hand, some dominant species may have low adaptive capacity because they are subject to other threats. For example, while the green ash-American elm assemblage was projected to experience increases in IV for dominant species overall, green ash is susceptible to emerald ash borer and American elm is susceptible to Dutch elm disease. Thus, the capacity of the assemblage to adapt to changing climate may be low, contributing to increased climate change vulnerability. Additional future threats that compromise adaptive capacity will come from urbanization, which is less dependent on the occurrence of a single species or species assemblage, and is likely to be high in the Northeast and in the Piedmont region of the Southeast [58,59].

As our case study demonstrates, because the underlying data contain all information about the species composition within each plot, and therefore within each cluster, the empirical typology of forest tree communities we identified can be used for a variety of assessment and monitoring purposes. The case study illustrates that metrics related to species dominance can inform an assessment of future climate change impacts to forest communities under the assumption that if dominant species are likely to experience substantial changes in suitable climate, the relative effects on the structure and function of the forest community will be high. A similar framework could be used to assess which communities and locations may be likely to experience impacts from pests or diseases using data such as the USDA Forest Service National Insect and Disease Risk Maps (https://www.fs.fed.us/foresthealth/technology/nidrm.shtml).

The empirical typology and associated data can also be the basis for projections of future change to forest communities. For example, community-level models that incorporate information on species co-occurrences to predict changes in the distributions of those co-occurrences have become popular recently as a way to examine potential future changes to forest communities [11,12,60,61]. Such models are currently in development and need improvement [62,63], but the empirical typology we developed, along with information about the relative occurrence of species within each cluster, would be well-suited for modeling in a community model framework. In fact, recent evidence shows that community models benefit from information about species dominance as a proxy for species interactions [16].

While the empirical typology of forest tree communities we identified can be used for a variety of monitoring and assessment purposes, a caveat in the analysis is worth noting. By excluding the rarest species in the inventory data, our clusters represent tree communities that are relatively common on the landscape. Because our analysis spanned the continental U.S., we necessarily sacrificed detail in local forest communities. The USNVC does include studies based on local plot data, especially at lower levels of the classification hierarchy, and complementary studies could also use FIA data across a smaller extent to delineate locally-specific communities.

## Conclusion

If evidence from past and recent responses to climate and land use change are any indication, future responses of tree communities to global change drivers are likely. Future changes to land use and climate will not only affect each tree species individually, but will have concomitant effects on forest communities as a whole. By identifying a hierarchy of forest tree communities and their associated indicator and dominant species, this work provides critical information that can be used to monitor changes and assess which communities and which locations might be most threatened by future change at a variety of extents.

## Supporting information

S1 Fig Dendrogram from hierarchical clustering of tree species importance values showing 147 specific assemblages and 29 broad assemblages. (PDF)

S2 Fig Change in importance values for dominant species in five broad assemblages. Maps show projected changes under the PCM Low scenario for plots in the (A) balsam fir-quaking aspen assemblage which had the largest projected decrease in importance value on average, (B) sugar maple-red maple assemblage, (C) loblolly pine-sweetgum assemblage, (D) slash pine-longleaf pine assemblage, and (E) green ash-American elm assemblage, which had the largest projected increase in importance value on average.
(PDF)

S1 Table Common and scientific names of all species that were included in cluster analysis. (PDF)

S2 Table Specific assemblages (147 clusters) corresponding to each broad assemblage (29 clusters). A blank line in the middle column indicates that there was more than one indicator species for the above specific assemblage.
(PDF)

S3 Table Dominant species for the broad assemblages (29 clusters) using the 85^th^ percentile cutoff to determine dominance. An indication of whether those species are dominant when the 90^th^ and 95^th^ percentile cutoffs are used is also shown.
(PDF)
