# Supplementary material for: An empirical, hierarchical typology of tree species assemblages for assessing forest dynamics under global change scenarios
Source: PLoS One. 2017 Sep 6;12(9):e0184062. doi: 10.1371/journal.pone.0184062 (PMC5587308; doi:10.1371/journal.pone.0184062)
Supplement: S1 Fig — (PDF) [file pone.0184062.s001.pdf]

Supplementary Material for

An empirical typology of tree species assemblages for assessing forest dynamics and threats

Jennifer K. Costanza, John W. Coulston, David N. Wear

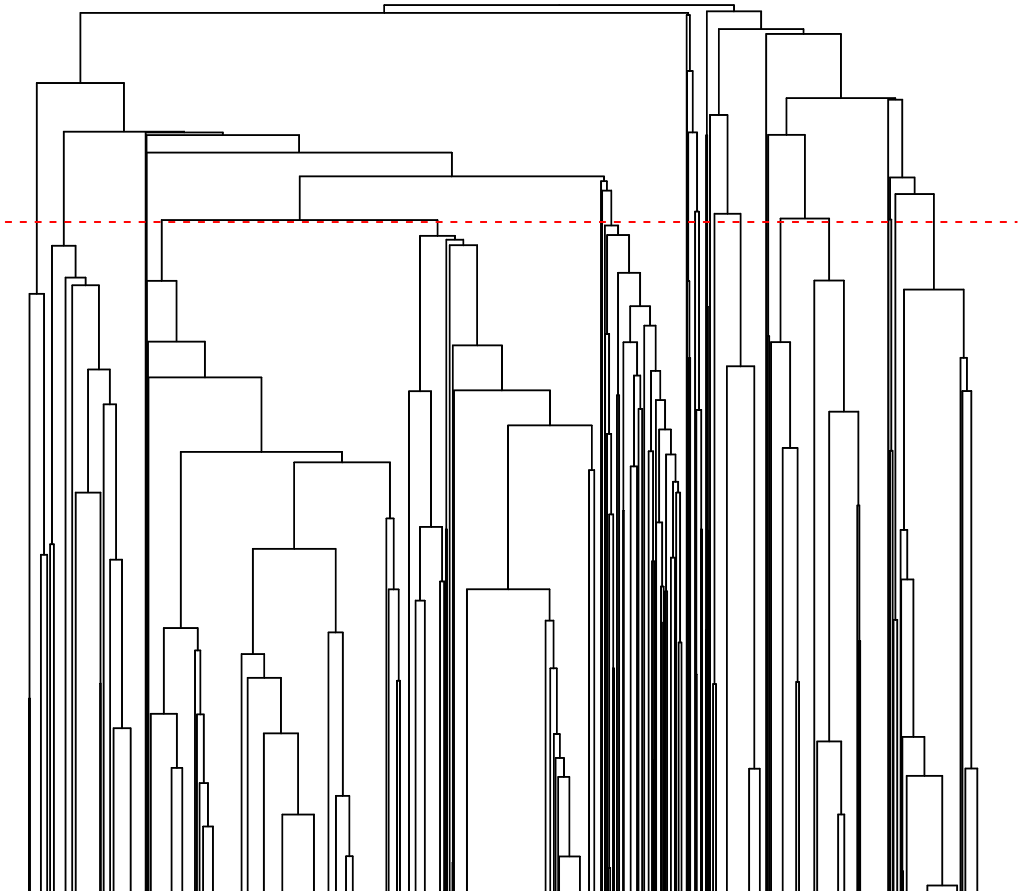

S1 Fig. Dendrogram from hierarchical clustering of tree species importance values showing 147 specific assemblages (at bottom), and 29 broad assemblages (dotted line).
