# Supplementary material for: An empirical, hierarchical typology of tree species assemblages for assessing forest dynamics under global change scenarios
Source: PLoS One. 2017 Sep 6;12(9):e0184062. doi: 10.1371/journal.pone.0184062 (PMC5587308; doi:10.1371/journal.pone.0184062)
Supplement: S2 Fig — Maps show projected changes under the PCM Low scenario for plots in the (A) balsam fir-quaking aspen assemblage which had the largest projected decrease in importance value on average, (B) sugar maple-red maple assemblage, (C) loblolly pine-sweetgum assemblage, (D) slash pine-longleaf pine assemblage, and (E) green ash-American elm assemblage, which had the largest projected increase in importance value on average. (PDF) [file pone.0184062.s002.pdf]

Supplementary Material for

An empirical typology of tree species assemblages for assessing forest dynamics and threats

Jennifer K. Costanza, John W. Coulston, David N. Wear

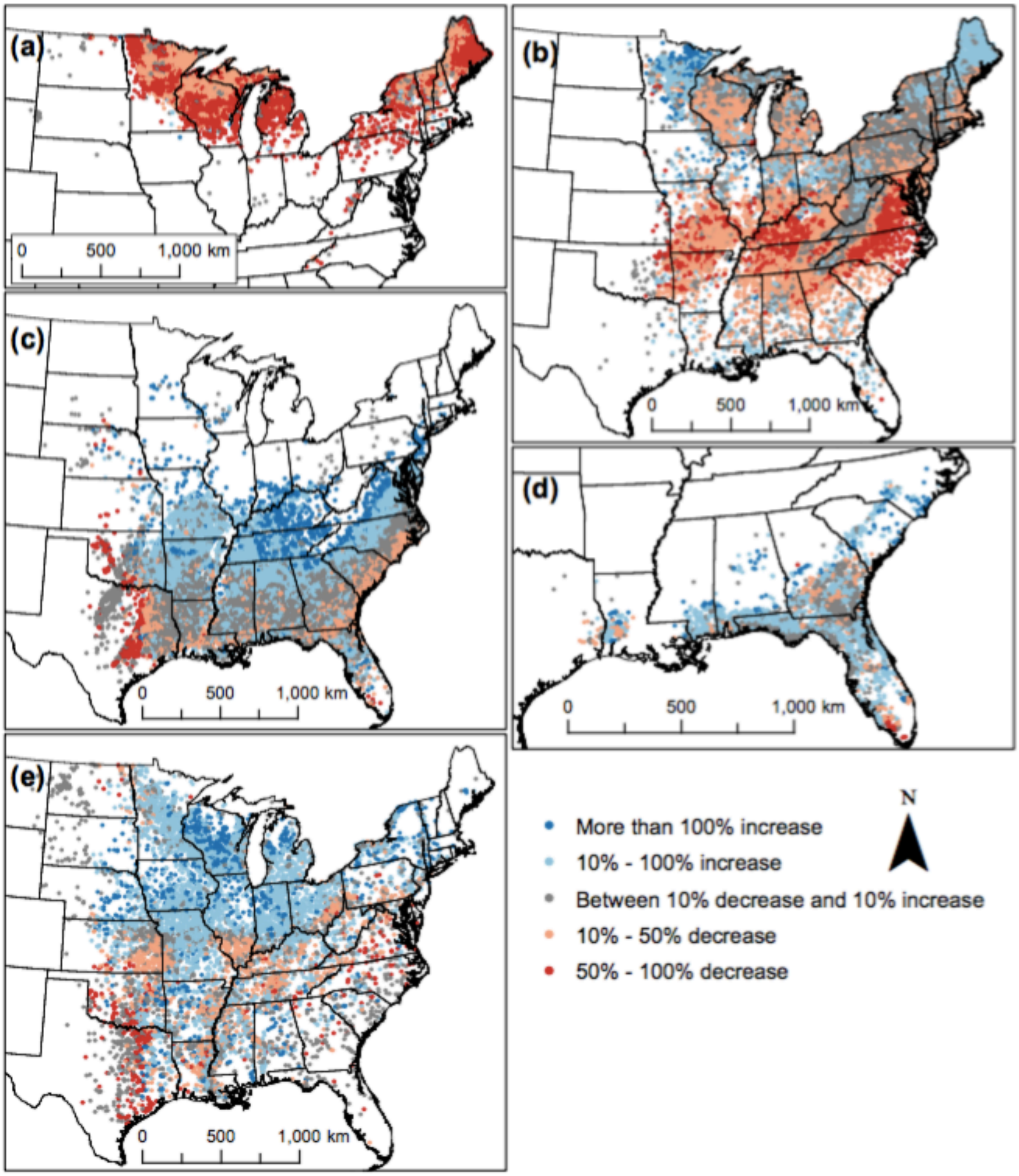

S2 Fig. Change in importance value for dominant species in five broad assemblages. Maps show projected changes under the PCM Low scenario for plots in the (A) balsam fir-quaking aspen assemblage which had the largest projected decrease in importance value on average, (B) sugar maple-red maple assemblage, (C) loblolly pine-sweetgum assemblage, (D) slash pine-

longleaf pine assemblage, and (E) green ash-American elm assemblage, which had the largest projected increase in importance value on average.
