# Supplementary material for: An empirical, hierarchical typology of tree species assemblages for assessing forest dynamics under global change scenarios
Source: PLoS One. 2017 Sep 6;12(9):e0184062. doi: 10.1371/journal.pone.0184062 (PMC5587308; doi:10.1371/journal.pone.0184062)
Supplement: S1 Table — (PDF) [file pone.0184062.s003.pdf]

Supporting information for

An empirical, hierarchical typology of tree species assemblages for assessing forest dynamics and threats

Jennifer K. Costanza, John W. Coulston, David N. Wear

S1 Table. Common and scientific names of all species that were included in cluster analysis.

Common name	Scientific name
alligator juniper	*Juniperus deppeana*
American basswood	*Tilia americana*
American beech	*Fagus grandifolia*
American elm	*Ulmus americana*
American holly	*Ilex opaca*
American hornbeam	*Carpinus caroliniana*
American plum	*Prunus americana*
American sycamore	*Platanus occidentalis*
Arizona white oak	*Quercus arizonica*
Ashe juniper	*Juniperus ashei*
baldcypress	*Taxodium distichum*
balsam fir	*Abies balsamea*
balsam poplar	*Populus balsamifera*
bigleaf maple	*Acer macrophyllum*
bigtooth aspen	*Populus grandidentata*
bitter cherry	*Prunus emarginata*
bitternut hickory	*Carya cordiformis*
black ash	*Fraxinus nigra*
black cherry	*Prunus serotina*
black cottonwood	*Populus balsamifera* ssp. *trichocarpa*
black hickory	*Carya texana*
black locust	*Robinia pseudoacacia*
black oak	*Quercus velutina*
black spruce	*Picea mariana*
black walnut	*Juglans nigra*
black willow	*Salix nigra*
blackgum	*Nyssa sylvatica*
blackjack oak	*Quercus marilandica*
blue oak	*Quercus douglasii*
boxelder	*Acer negundo*
bur oak	*Quercus macrocarpa*

butternut	*Juglans cinerea*
cabbage palmetto	*Sabal palmetto*
California black oak	*Quercus kelloggii*
California laurel	*Umbellularia californica*
California live oak	*Quercus agrifolia*
California red fir	*Abies magnifica*
canyon live oak	*Quercus chrysolepis*
cedar elm	*Ulmus crassifolia*
cherrybark oak	*Quercus falcata* var. *pagodaefolia*
chestnut oak	*Quercus prinus*
chinkapin oak	*Quercus muehlenbergii*
chittamwood	*Bumelia lanuginosa* (*Sideroxylon lanuginosum* ssp. *Lanuginosum*)
chokecherry	*Prunus virginiana*
coast Douglas-fir	*Pseudotsuga menziesii* var. *menziesii*
common persimmon	*Diospyros virginiana*
cucumbertree	*Magnolia acuminata*
curlleaf mountain-mahogany	*Cercocarpus ledifolius*
eastern cottonwood	*Populus deltoides*
eastern hemlock	*Tsuga canadensis*
eastern hophornbeam	*Ostrya virginiana*
eastern redbud	*Cercis canadensis*
eastern redcedar	*Juniperus virginiana*
eastern white pine	*Pinus strobus*
Emory oak	*Quercus emoryi*
Engelmann spruce	*Picea engelmannii*
Florida maple	*Acer barbatum*
flowering dogwood	*Cornus florida*
Gambel oak	*Quercus gambelii*
giant chinkapin	*Chrysolepis chrysophylla* var. *chrysophylla*
grand fir	*Abies grandis*
gray birch	*Betula populifolia*
gray pine	*Pinus sabiniana*
green ash	*Fraxinus pennsylvanica*
hackberry	*Celtis occidentalis*
hawthorn spp.	*Crataegus* spp.
honey mesquite	*Prosopis glandulosa*

honeylocust	*Gleditsia triacanthos*
incense-cedar	*Calocedrus decurrens*
interior live oak	*Quercus wislizeni*
jack pine	*Pinus banksiana*
Jeffrey pine	*Pinus jeffreyi*
laurel oak	*Quercus laurifolia*
limber pine	*Pinus flexilis*
live oak	*Quercus virginiana*
loblolly pine	*Pinus taeda*
loblolly-bay	*Gordonia lasianthus*
lodgepole pine	*Pinus contorta*
longleaf pine	*Pinus palustris*
mockernut hickory	*Carya tomentosa*
mountain hemlock	*Tsuga mertensiana*
mountain magnolia	*Magnolia fraseri*
mountain maple	*Acer spicatum*
noble fir	*Abies procera*
northern pin oak	*Quercus ellipsoidalis*
northern red oak	*Quercus rubra*
northern white-cedar	*Thuja occidentalis*
Ohio buckeye	*Aesculus glabra*
oneseed juniper	*Juniperus monosperma*
Oregon white oak	*Quercus garryana*
Osage-orange	*Maclura pomifera*
overcup oak	*Quercus lyrata*
Pacific dogwood	*Cornus nuttallii*
Pacific madrone	*Arbutus menziesii*
Pacific silver fir	*Abies amabilis*
Pacific yew	*Taxus brevifolia*
paper birch	*Betula papyrifera*
pawpaw	*Asimina triloba*
pecan	*Carya illinoensis*
pignut hickory	*Carya glabra*
pin cherry	*Prunus pensylvanica*
pin oak	*Quercus palustris*
Pinchot juniper	*Juniperus pinchotii*
pitch pine	*Pinus rigida*
pond pine	*Pinus serotina*
pondcypress	*Taxodium distichum*

ponderosa pine	*Pinus ponderosa*
post oak	*Quercus stellata*
quaking aspen	*Populus tremuloides*
red alder	*Alnus rubra*
red maple	*Acer rubrum*
red mulberry	*Morus rubra*
red pine	*Pinus resinosa*
red spruce	*Picea rubens*
redbay	*Persea borbonia*
redberry juniper	*Juniperus coahuilensis*
redwood	*Sequoia sempervirens*
river birch	*Betula nigra*
Rocky Mountain Douglas-fir	*Pseudotsuga menziesii* var. *glauca*
Rocky Mountain juniper	*Juniperus scopulorum*
Rocky Mountain maple	*Acer glabrum*
sassafras	*Sassafras albidum*
scarlet oak	*Quercus coccinea*
serviceberry spp.	*Amelanchier* spp.
shagbark hickory	*Carya ovata*
shellbark hickory	*Carya laciniosa*
shingle oak	*Quercus imbricaria*
shortleaf pine	*Pinus echinata*
Shumard oak	*Quercus shumardii*
silver maple	*Acer saccharinum*
singleleaf pinyon	*Pinus monophylla*
Sitka spruce	*Picea sitchensis*
slash pine	*Pinus elliottii*
slippery elm	*Ulmus rubra*
sourwood	*Oxydendrum arboreum*
southern magnolia	*Magnolia grandiflora*
southern red oak	*Quercus falcata* var. *falcata*
spruce pine	*Pinus glabra*
striped maple	*Acer pensylvanicum*
subalpine fir	*Abies lasiocarpa*
sugar maple	*Acer saccharum*
sugar pine	*Pinus lambertiana*
sugarberry	*Celtis laevigata*
swamp chestnut oak	*Quercus michauxii*
swamp tupelo	*Nyssa biflora*

swamp white oak	*Quercus bicolor*
sweet birch	*Betula lenta*
sweetbay	*Magnolia virginiana*
sweetgum	*Liquidambar styraciflua*
tamarack (native)	*Larix laricina*
tanoak	*Lithocarpus densiflorus*
Texas persimmon	*Diospyros texana*
Texas red oak	*Quercus nuttallii*
turkey oak	*Quercus laevis*
two needle pinyon	*Pinus edulis*
Utah juniper	*Juniperus osteosperma*
velvet mesquite	*Prosopis velutina*
Virginia pine	*Pinus virginiana*
water hickory	*Carya aquatica*
water oak	*Quercus nigra*
water tupelo	*Nyssa aquatica*
western hemlock	*Tsuga heterophylla*
western juniper	*Juniperus occidentalis*
western larch	*Larix occidentalis*
western redcedar	*Thuja plicata*
western white pine	*Pinus monticola*
white ash	*Fraxinus americana*
white fir	*Abies concolor*
white oak	*Quercus alba*
white spruce	*Picea glauca*
whitebark pine	*Pinus albicaulis*
willow oak	*Quercus phellos*
winged elm	*Ulmus alata*
yellow birch	*Betula alleghaniensis*
yellow buckeye	*Aesculus octandra*
yellow-poplar	*Liriodendron tulipifera*
