# Supplementary material for: An empirical, hierarchical typology of tree species assemblages for assessing forest dynamics under global change scenarios
Source: PLoS One. 2017 Sep 6;12(9):e0184062. doi: 10.1371/journal.pone.0184062 (PMC5587308; doi:10.1371/journal.pone.0184062)
Supplement: S2 Table — A blank line in the middle column indicates that there was more than one indicator species for the above specific assemblage. (PDF) [file pone.0184062.s004.pdf]

Supporting information for

An empirical, hierarchical typology of tree species assemblages for assessing forest dynamics and threats

Jennifer K. Costanza, John W. Coulston, David N. Wear

S2 Table. Specific assemblages (147 clusters) corresponding to each broad assemblage (29 clusters). A blank line in the middle column indicates that there was more than one indicator species for the above specific assemblage.

Broad assemblage	Num. plots in specific assemblage	Indicator species for specific assemblage
**slash pine-longleaf pine**	1867	slash pine
	718	longleaf pine
	259	pondcypress
	90	turkey oak
**balsam fir-quaking aspen**	2790	balsam fir
	4122	quaking aspen
	1530	black spruce
		tamarack (native)
	918	black ash
	43	gray birch
	1112	red pine
	1118	northern white cedar
	214	white spruce
	278	paper birch
	472	jack pine
	16	balsam poplar
		mountain maple
		yellow birch
**common persimmon**	83	common persimmon
**butternut-sweet birch**	6	butternut
**sourwood-scarlet oak**	6	scarlet oak
		sourwood
**sugar maple-red maple**	4902	sugar maple
	865	black oak
	1422	American beech
		striped maple
	3335	white oak
	1825	eastern white pine
	3726	red maple
	687	white ash
	130	sassafras

	1954	yellow poplar
	1713	northern red oak
	2529	eastern hemlock
		sweet birch
	2733	chestnut oak
	826	Virginia pine
	1197	black cherry
	561	American basswood
	363	pignut hickory
	619	bigtooth aspen
	165	pitch pine
	317	mockernut hickory
	67	Florida maple
	12	serviceberry spp .
	52	pin cherry
	63	yellow buckeye
	5	pawpaw
**loblolly pine-sweetgum**	12796	loblolly pine
	503	baldcypress
		water tupelo
	1177	shortleaf pine
	3599	sweetgum
	428	southern red oak
	197	blackgum
	174	American holly
	1441	eastern redcedar
	2127	black hickory
		blackjack oak
		post oak
	134	river birch
	750	water oak
	1007	swamp tupelo
		sweetbay
	366	laurel oak
	53	redbay
	68	flowering dogwood
	359	willow oak
	91	cherrybark oak
	83	loblolly bay
	41	eastern redbud
	127	pond pine
	54	southern magnolia
		spruce pine

**hawthorn spp.-American plum**	88	hawthorn spp.
**black willow**	343	black willow
**green ash-American elm**	666	hackberry
	318	eastern cottonwood
	39	Shumard oak
	523	boxelder
	276	northern pin oak
	222	honeylocust
	528	silver maple
	386	American sycamore
	112	pin oak
	671	American elm
	512	shagbark hickory
	57	Texas red oak
	77	chinkapin oak
		Ohio buckeye
	428	black locust
	481	black walnut
	311	Osage orange
	437	sugarberry
	101	red mulberry
	1191	green ash
	183	winged elm
	98	slippery elm
	133	shingle oak
	200	eastern hophornbeam
	69	swamp chestnut oak
	606	bur oak
	155	pecan
	164	bitternut hickory
	82	American hornbeam
	223	overcup oak
		water hickory
	6	American plum
	35	swamp white oak
	13	shellbark hickory
**velvet mesquite**	269	velvet mesquite
**chittamwood**	18	chitthamwood
**honey mesquite-Pinchot juniper**	265	redberry juniper
	3041	honey mesquite
	455	Pinchot juniper
**cedar elm**	149	cedar elm

**live oak-Ashe juniper**	61	Texas persimmon
	780	live oak
	766	Ashe juniper
	183	cabbage palmetto
**California live oak-California laurel**	59	California laurel
	165	California live oak
**blue oak-interior live oak**	195	interior live oak
	351	blue oak
	39	gray pine
**Gambel oak**	769	Gambel oak
**alligator juniper-Arizona white oak**	643	alligator juniper
		Arizona white oak
	93	Emory oak
**Utah juniper-two needle pinyon**	1682	oneseed juniper
	5020	singleleaf pinyon
		Utah juniper
	1087	two needle pinyon
**black cottonwood-bigleaf maple**	44	bigleaf maple
		black cottonwood
**western juniper-curlleaf mountain mahogany**	351	curlleaf mountain mahogany
	817	western juniper
**lodgepole pine-subalpine fir**	3058	Engelmann spruce
		subalpine fir
	2732	lodgepole pine
	69	western white pine
	210	whitebark pine
**Rocky Mountain Douglas-fir-ponderosa pine**	5337	ponderosa pine
	3374	Rocky Mountain Douglas-fir
	572	Rocky Mountain juniper
	1806	grand fir
		western redcedar
	387	western larch
	105	limber pine
	4	Rocky Mountain maple
**chokecherry-Pacific dogwood**	13	chokecherry
		Pacific dogwood
**Oregon white oak**	213	Oregon white oak
**canyon live oak-California black oak**	125	California black oak
	284	canyon live oak
**mountain hemlock-Pacific silver fir**	815	mountain hemlock
		Pacific silver fir

	104	noble fir
**coast Douglas-fir-western hemlock**	7065	coast Douglas-fir
	272	California red fir
	300	Jeffrey pine
	1421	white fir
	565	giant chinkapin
		redwood
		tanoak
	321	incense cedar
		sugar pine
	73	Pacific madrone
	65	red alder
		Sitka spruce
		western hemlock
	11	bitter cherry
