# Supplementary material for: An empirical, hierarchical typology of tree species assemblages for assessing forest dynamics under global change scenarios
Source: PLoS One. 2017 Sep 6;12(9):e0184062. doi: 10.1371/journal.pone.0184062 (PMC5587308; doi:10.1371/journal.pone.0184062)
Supplement: S3 Table — An indication of whether those species are dominant when the 90th and 95th percentile cutoffs are used is also shown. (PDF) [file pone.0184062.s005.pdf]

Supplementary Material for
An empirical typology of tree species assemblages for assessing forest dynamics and threats
Jennifer K. Costanza, John W. Coulston, David N. Wear

S3 Table. Dominant species for the broad assemblages (29 clusters) using the 85th percentile cutoff to determine dominance. An indication of whether those species are dominant when the 90th and 95th percentile cutoffs are used is also shown.

Broad assemblage	Dominant species: 85th percentile	SDI	Also in 90th percentile of dominance?	Also in 95th percentile of dominance?
**slash pine-longleaf pine**	slash pine	0.58	Yes	Yes
	longleaf pine	0.32	Yes	Yes
	pondcypress	0.22	Yes	Yes
	turkey oak	0.13	Yes	No
	chestnut oak	0.07	No	No
**balsam fir-quaking aspen**	quaking aspen	0.32	Yes	Yes
	red pine	0.24	Yes	Yes
	northern white cedar	0.18	Yes	Yes
	balsam fir	0.17	Yes	No
	black spruce	0.16	Yes	No
	jack pine	0.16	Yes	No
	tamarack (native)	0.13	Yes	No
	black ash	0.12	Yes	No
	Jeffrey pine	0.11	Yes	No
	red spruce	0.10	Yes	No
	California red fir	0.08	No	No
	western redcedar	0.08	No	No
	lodgepole pine	0.07	No	No
	paper birch	0.07	No	No
	black cottonwood	0.07	No	No
	yellow birch	0.06	No	No
	balsam poplar	0.06	No	No
**common persimmon**	common persimmon	0.71	Yes	Yes
	black cherry	0.11	Yes	No
	black willow	0.10	No	No
**butternut-sweet birch**	butternut	0.81	Yes	Yes
**sourwood-scarlet oak**	sourwood	0.74	Yes	Yes
	scarlet oak	0.06	No	No
**sugar maple-red maple**	sugar maple	0.17	Yes	No
	red maple	0.17	Yes	No
	eastern white pine	0.12	Yes	No
	white oak	0.12	Yes	No

	chestnut oak	0.11	Yes	No
	yellow poplar	0.11	Yes	No
	eastern hemlock	0.11	Yes	No
	Virginia pine	0.10	Yes	No
	northern red oak	0.08	No	No
	pitch pine	0.08	No	No
	American beech	0.08	No	No
	bigtooth aspen	0.07	No	No
	black cherry	0.07	No	No
	black oak	0.07	No	No
	Rocky Mountain Douglas-fir	0.07	No	No
**loblolly pine-sweetgum**	loblolly pine	0.40	Yes	Yes
	water tupelo	0.15	Yes	No
	pond pine	0.14	Yes	No
	sweetgum	0.13	Yes	No
	post oak	0.12	Yes	No
	eastern redcedar	0.12	Yes	No
	baldcypress	0.12	Yes	No
	shortleaf pine	0.10	Yes	No
	swamp tupelo	0.10	Yes	No
	loblolly bay	0.10	Yes	No
	water oak	0.08	No	No
	laurel oak	0.07	No	No
**hawthorn spp.-American plum**	hawthorn spp.	0.65	Yes	Yes
	eastern hophornbeam	0.18	Yes	Yes
	black willow	0.18	Yes	Yes
	blackgum	0.17	Yes	Yes
	American elm	0.13	Yes	No
	shagbark hickory	0.13	Yes	No
	American plum	0.12	Yes	No
	serviceberry spp.	0.12	Yes	No
	overcup oak	0.09	No	No
	sugar maple	0.07	No	No
**black willow**	black willow	0.65	Yes	Yes
	green ash	0.13	Yes	No
	eastern redbud	0.11	Yes	No
	silver maple	0.09	No	No
	red maple	0.08	No	No
	swamp tupelo	0.08	No	No
	yellow poplar	0.07	No	No
	sweetgum	0.06	No	No

**green ash-American elm**	silver maple	0.19	Yes	Yes
	northern pin oak	0.19	Yes	Yes
	green ash	0.17	Yes	No
	eastern cottonwood	0.16	Yes	No
	bur oak	0.16	Yes	No
	black locust	0.15	Yes	No
	boxelder	0.13	Yes	No
	sugarberry	0.12	Yes	No
	Osage orange	0.12	Yes	No
	American elm	0.11	Yes	No
	hackberry	0.11	Yes	No
	American sycamore	0.11	Yes	No
	shagbark hickory	0.11	Yes	No
	pecan	0.10	Yes	No
	pin oak	0.10	Yes	No
	black walnut	0.10	Yes	No
	overcup oak	0.10	Yes	No
	honeylocust	0.09	No	No
	Texas red oak	0.09	No	No
	winged elm	0.08	No	No
	shingle oak	0.08	No	No
	swamp white oak	0.07	No	No
	ponderosa pine	0.07	No	No
	water hickory	0.07	No	No
	bitternut hickory	0.06	No	No
	Shumard oak	0.06	No	No
**velvet mesquite**	velvet mesquite	0.97	Yes	Yes
	redberry juniper	0.10	Yes	No
**chittamwood**	chittamwood	0.90	Yes	Yes
**honey mesquite-Pinchot juniper**	honey mesquite	0.81	Yes	Yes
	Pinchot juniper	0.34	Yes	Yes
	redberry juniper	0.32	Yes	Yes
	oneseed juniper	0.17	Yes	Yes
	Ashe juniper	0.08	No	No
**cedar elm**	cedar elm	0.64	Yes	Yes
	willow oak	0.20	Yes	Yes
	water oak	0.15	Yes	No
	Osage orange	0.15	Yes	No
	eastern redcedar	0.11	Yes	No
**live oak-Ashe juniper**	Ashe juniper	0.53	Yes	Yes
	live oak	0.43	Yes	Yes
	cabbage palmetto	0.29	Yes	Yes

	Texas persimmon	0.11	Yes	No
**California live oak-California laurel**	California live oak	0.73	Yes	Yes
	California laurel	0.32	Yes	Yes
	blue oak	0.13	Yes	No
	red alder	0.12	Yes	No
**blue oak-interior live oak**	blue oak	0.65	Yes	Yes
	interior live oak	0.38	Yes	Yes
	Pacific dogwood	0.33	Yes	Yes
	gray or California foothill pine	0.20	Yes	Yes
	singleleaf pinyon	0.14	Yes	No
	canyon live oak	0.09	No	No
**Gambel oak**	Gambel oak	0.82	Yes	Yes
	quaking aspen	0.08	No	No
	white fir	0.08	No	No
**alligator juniper-Arizona white oak**	alligator juniper	0.41	Yes	Yes
	Arizona white oak	0.31	Yes	Yes
	Emory oak	0.21	Yes	Yes
	ponderosa pine	0.14	Yes	No
	Gambel oak	0.09	No	No
	common or two needle pinyon	0.09	No	No
	velvet mesquite	0.08	No	No
	Utah juniper	0.06	No	No
**Utah juniper-two needle pinyon**	Utah juniper	0.49	Yes	Yes
	oneseed juniper	0.35	Yes	Yes
	singleleaf pinyon	0.29	Yes	Yes
	common or two needle pinyon	0.28	Yes	Yes
	western juniper	0.08	No	No
	redberry juniper	0.07	No	No
	Gambel oak	0.07	No	No
	Jeffrey pine	0.06	No	No
**black cottonwood-bigleaf maple**	black cottonwood	0.86	Yes	Yes
	bigleaf maple	0.20	Yes	Yes
**western juniper-curlleaf mountain mahogany**	western juniper	0.69	Yes	Yes
	curlleaf mountain mahogany	0.39	Yes	Yes
	ponderosa pine	0.17	Yes	No

	Rocky Mountain juniper	0.07	No	No
**lodgepole pine-subalpine fir**	lodgepole pine	0.46	Yes	Yes
	subalpine fir	0.31	Yes	Yes
	Engelmann spruce	0.26	Yes	Yes
	whitebark pine	0.14	Yes	No
	western white pine	0.09	No	No
	Rocky Mountain Douglas-fir	0.07	No	No
**Rocky Mountain Douglas-fir-ponderosa pine**	ponderosa pine	0.46	Yes	Yes
	Rocky Mountain Douglas-fir	0.36	Yes	Yes
	grand fir	0.20	Yes	Yes
	Rocky Mountain juniper	0.17	Yes	No
	western redcedar	0.14	Yes	No
	interior live oak	0.09	No	No
	limber pine	0.09	No	No
	bur oak	0.08	No	No
	western larch	0.08	No	No
	lodgepole pine	0.08	No	No
	coast Douglas-fir	0.06	No	No
**chokecherry-Pacific dogwood**	chokecherry	0.75	Yes	Yes
**Oregon white oak**	Oregon white oak	0.74	Yes	Yes
	ponderosa pine	0.10	Yes	No
	Pacific madrone	0.07	No	No
**canyon live oak-California black oak**	canyon live oak	0.57	Yes	Yes
	California black oak	0.36	Yes	Yes
	blue oak	0.07	No	No
**mountain hemlock-Pacific silver fir**	Pacific silver fir	0.42	Yes	Yes
	mountain hemlock	0.41	Yes	Yes
	noble fir	0.19	Yes	Yes
	Pacific yew	0.11	Yes	No
	whitebark pine	0.07	No	No
	subalpine fir	0.07	No	No
**coast Douglas-fir-western hemlock**	coast Douglas-fir	0.45	Yes	Yes
	white fir	0.22	Yes	Yes
	redwood	0.20	Yes	Yes
	California red fir	0.18	Yes	Yes

Jeffrey pine	0.18	Yes	Yes
western hemlock	0.17	Yes	Yes
tanoak	0.13	Yes	No
red alder	0.10	Yes	No
Rocky Mountain Douglas-fir	0.08	No	No
incense cedar	0.07	No	No
ponderosa pine	0.07	No	No
Pacific silver fir	0.06	No	No
